# Ultrasonographic, clinical, and pathological features of papillary thyroid carcinoma in children and adolescents with or without Hashimoto’s thyroiditis

**DOI:** 10.3389/fonc.2023.1198468

**Published:** 2023-08-01

**Authors:** Yue Jie, Jingliang Ruan, Man Luo, Rongbin Liu

**Affiliations:** ^1^ Department of Ultrasound, Sun Yat-sen Memorial Hospital, Sun Yat-sen University, Guangzhou, China; ^2^ GuangDong Provincial Key Laboratory of Malignant Tumor Epigenetics and Gene Regulation, Sun Yat-sen Memorial Hospital, Sun Yat-sen University, Guangzhou, China

**Keywords:** Hashimoto’s thyroiditis, papillary thyroid carcinoma, ultrasonographic features, histopathological features, children and adolescents

## Abstract

**Objective:**

To compare the ultrasonographic, clinical, and pathological features of children and adolescents with papillary thyroid carcinoma (PTC) with and without Hashimoto’s thyroiditis (HT)

**Materials and methods:**

A total of 52 children and adolescent patients surgically diagnosed with PTC between 2017 and 2022 were included; 14 children and adolescent patients with PTC were diagnosed with HT *via* pathological examination. The preoperative ultrasonographic, postoperative histological, and molecular and clinical characteristics were retrospectively analyzed.

**Results:**

The prevalence rate of PTC in patients with HT was 27%. Papillary thyroid microcarcinomas were found in 11 of 38 patients without HT, but none in patients with HT (p = 0.023). Extrathyroidal extension, capsular invasion, and lymph node metastases were more frequent in patients with PTC and HT than in patients with PTC alone (p < 0.05 for both). The ultrasonographic features of nodule composition, echogenicity, shape, margin, Thyroid Imaging Reporting and Data System categories, and total points were similar. The patterns of echogenic foci were more prominent in the nodules of patients with HT than in those of patients without HT (p = 0.016).

**Conclusion:**

The frequency of papillary thyroid microcarcinomas in patients with PTC and HT was less, whereas that of extrathyroidal extension, capsular invasion, and lymph node metastasis was significantly higher in patients with PTC and HT than in those with PTC alone. The patterns of echogenic foci on ultrasonography may represent a risk for PTC.

## 1 Introduction

Thyroid cancer is rare in children and adolescents; however, the incidence appears to be increasing in recent years (1, 2). According to the Surveillance, Epidemiology, and End Results database, the incidence of thyroid cancer in children and adolescents aged ≤18 years increased by an average of 4.43% per year during 1998–2013 in the United States (3, 4). Papillary thyroid carcinoma (PTC)is the most common one, accounting for more than 90% of all children and adolescent cases; the incidence of PTC peaks between the ages of 15 and 18 years (5).

Hashimoto’s thyroiditis (HT) is an organ-specific autoimmune disease and the most common cause of hypothyroidism in iodine-sufficient areas; it is characterized by diffuse lymphocytic infiltration and affects 1.3%–9.6% of children and adolescents (6–8).

Whether HT is a risk factor for PTC remains unclear (9). Many studies have shown a higher risk of PTC in patients with HT (10–12), whereas others did not demonstrate the increased risk (13, 14). PTC intertwined with HT in children and adolescents is extremely rare; few studies have assessed the association between PTC and HT in children and adolescents.

In the present study, we aimed to compare the preoperative ultrasonographic, clinical, and pathological features of children and adolescents with PTC with and without HT.

## 2 Patients and methods

### 2.1 Study design and patients

Approval for this retrospective study was obtained from the Institutional Research Ethics Board of Sun Yat-sen Memorial Hospital, Sun Yat-sen University, Guangzhou, China; the requirement for informed patient consent was waived.

The medical records of our institution were searched between January 2017 and 2022; 52 patients (mean age: 14 years, range: 4–18 years) who underwent thyroid cancer surgery and lateral cervical lymph node (LN) dissection were included.

The medical records of all subjects were retrospectively reviewed, and patient demographics (age and sex), ultrasonographic features [composition, echogenicity, shape, margin, echogenic foci, total points, and TI-RADS level based on the American College of Radiology Thyroid Imaging Reporting and Data System (ACR TI-RADS) classification (15), LN metastasis (LNM)], pathological findings (primary tumor size, HT, multifocal tumor, extrathyroidal extensions, capsular invasion, LNM, BRAF^V600E^ mutation status), and TNM stage were analyzed. PTC was confirmed by pathological findings and staged according to the 8th edition of the Union for International Cancer Control/American Joint Committee on Cancer TNM staging system for differentiated and anaplastic thyroid carcinoma (16).

### 2.2 Ultrasound examination of thyroid nodules

Real-time ultrasonography and Doppler examinations were performed using Resona 7 (Mindray medical system, Shenzhen, China) equipped with L14-5 WU linear array transducer (5–14 MHz); ACUSON Sequoia (Siemens Healthcare, Erlangen, Germany) equipped with 18L6 linear array transducer (6–18 MHz); ALOKA Arietta 850 premium (FUJIFILM Corporation, Tokyo, Japan) equipped with SML2-22 linear array probe (2–22 MHz) by three radiologists with more than 5 years of experience in thyroid ultrasonography.

According to the 2017 ACR TI-RADS, the five categories for ultrasound appearance of all thyroid nodules were analyzed: (1) composition: cystic, mixed, or solid or almost completely solid; (2) echogenicity: hypoechoic, isoechoic, or hyperechoic; (3) shape: wider-than-tall or taller-than-wide; (4) margin: smooth, ill-defined, lobulated, or irregular or extra-thyroidal extension; (5) echogenic foci: none or large comet-tail artifacts, macrocalcifications, punctate echogenic foci (PEF). Moreover, the five categories from the ACR-TIRADS 2017 point were registered. The total point of each nodule was added to determine the TI-RADS level. Furthermore, the LNs were considered metastatic if their shortest diameter exceeded 7 mm in levels I and II or 6 mm in levels III, IV, and V, and the ratio of their shortest to longest diameters exceeded 0.5 mm concurrently. In case of any discrepancy, the final ACR TI-RADS point scores and specific ACR TI-RADS classification were taken as the average with an agreement after discussion.

### 2.3 Pathology examination

The patients underwent thyroidectomy (lobectomy, near total thyroidectomy, total thyroidectomy) with regional lymphadenectomy (central neck dissection, lateral neck dissection, superior mediastinal dissection, or a combination of the above). The selection of all surgical methods is based on the American Thyroid Association guidelines for pediatric thyroid nodules (5) and Chinese expert consensus on thyroid nodules and differentiated thyroid cancer for Chinese children (5). The thyroid gland and LN histological sections were stained with hematoxylin and eosin staining, and the histological slides were independently evaluated by two senior pathologists using a double-blind method. The patients were diagnosed with HT based on the presence of diffuse lymphocytic and plasma cell infiltration, oxyphilic cells, and lymphoid follicles with reactive germinal centers.

The baseline information, including age, sex, primary tumor size, diagnosis of HT, number of lesions, pathological type, LNM, extrathyroidal extensions, capsular invasion, BRAF^V600E^ mutation status, and TNM stage, was collected. All patients were staged using the thyroid cancer staging system released by the American Joint Committee on Cancer (8th ed., 2017).

### 2.4 Statistical analysis

All statistical analyses were performed using SPSS version 22.0 (SPSS Inc., Chicago, IL, USA). Descriptive analyses were expressed as mean ± standard deviation (X ± SD) for normally distributed variables, median (range) or median (interquartile range) for non-normally distributed variables, and the interquartile range for categorized variables. The student’s t-test, Mann–Whitney U-test, or χ^2^ test were used to assess the heterogeneity of demographic characteristics between groups with or without HT. P < 0.05 was considered statistically significant.

## 3 Results

### 3.1 Clinical features and pathological findings

A total of 52 children and adolescent patients were included. They were divided into the HT group (n = 14, mean age = 15.07 ± 2.62 years; 1 male and 13 female) and the non-HT group (n = 38, mean age = 14.34 ± 3.32 years; 9 male and 29 female). The comparison of demographics and pathological findings of PTC between the two groups is summarized in **Table 1**. All patients underwent thyroidectomy with regional lymphadenectomy. In the HT group, 7 patients underwent lobectomy, 2 patients underwent near thyroidectomy, 5 patients underwent total thyroidectomy. In the non-HT group, 18 patients underwent lobectomy, 6 patients underwent near thyroidectomy, 14 patients underwent total thyroidectomy; the final pathology was PTC in all cases.

**Table 1:** Characteristics between children and adolescents’ patients with or without Hashimoto’s thyroiditis (HT).

Characteristics	PTC with TH	PTC without TH	P value
Number of patients	14(26.92%)	38(73.08%)	
Years			0.356
Mean ± SD	15.07 ± 2.62	14.34 ± 3.32	
Range	8-18	4-18	
Gender			0.179
Female	13(25.00%)	29(55.77%)	
Male	1(1.92%)	9(17.31%)	
Primary tumor size (cm)			0.023
< 1	0	11(21.15%)	
≥ 1	14(26.92%)	27(51.92%)	
Mean ± SD	22.00 ± 8.89	23.76 ± 17.73	0.725
Range	13-45	3-80	
Multifocal tumor			0.864
Yes	3(57.69%)	9(17.31%)	
No	11(21.15%)	29(55.77%)	
Extrathyroidal extension			0.013
Yes	12(23.08%)	18(34.62%)	
No	2(3.85%)	20(38.46%)	
Capsular invasion			0.023
Yes	14(26.92%)	27(51.92%)	
No	0	11(21.15%)	
T staging			0.323
T1	8(15.38%)	13(25.00%)	
T2	5(9.62%)	20(38.46%)	
T3	1(1.92%)	5(9.62%)	
N staging			0.239
N0	1(1.92%)	11(21.15%)	
N1a	7(13.46%)	13(25.00%)	
N1b	6(11.54%)	14(26.92%)	
BRAF^V600E^ mutation			0.715
Positive	4(7.69%)	13(25.00%)	
Negative	8(15.38%)	17(32.69%)	
Not detected	2(3.85%)	8(15.38%)	

**Figure 1:**
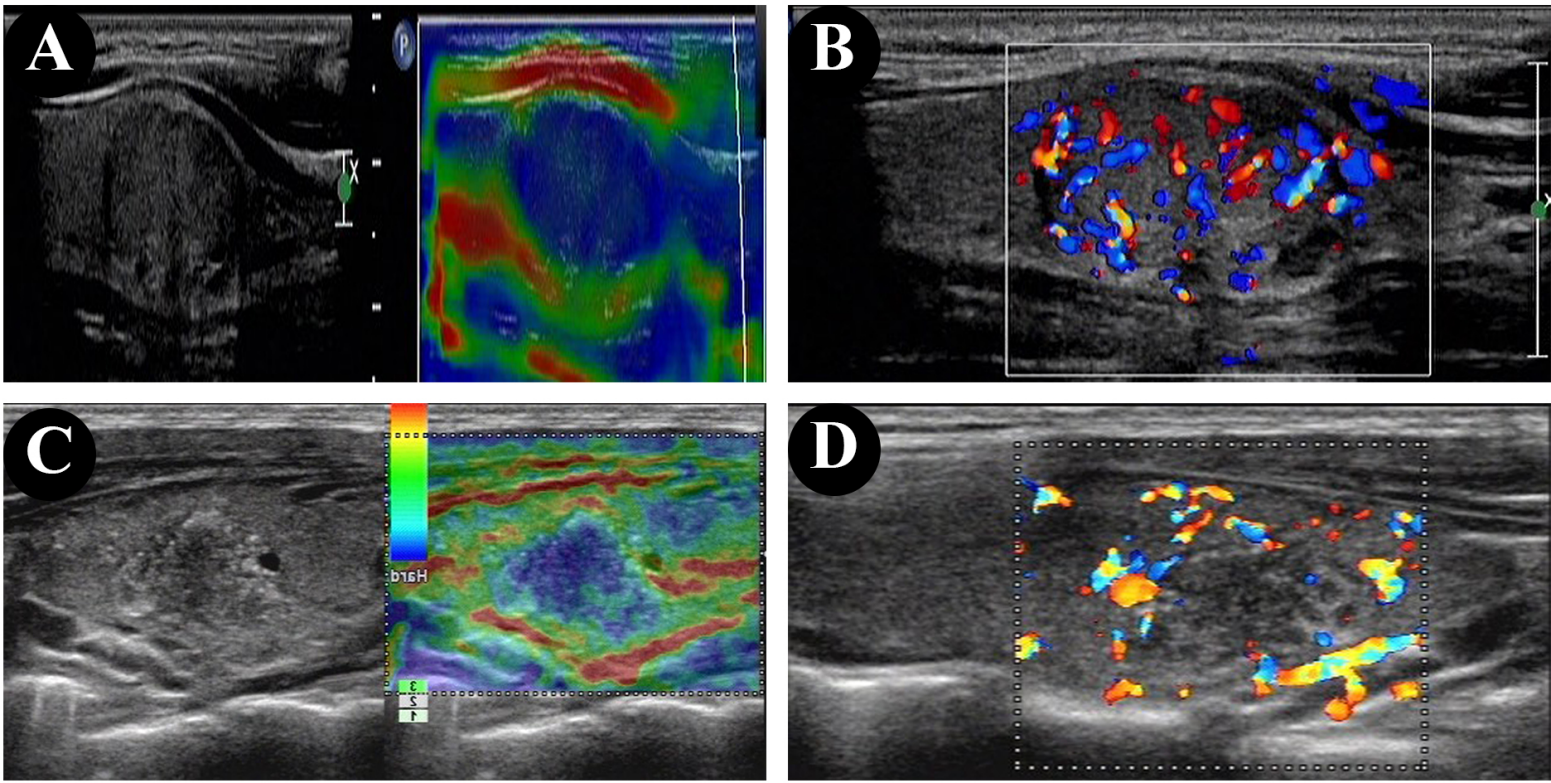
Ultrasonographic and elastography findings of PTC showed the tumor is closely attached to the capsule in the patient without HT **(A, B)**, and extrathyroidal extension in the patient with HT **(C, D)**.

There were 11 papillary thyroid microcarcinomas (≤1 cm diameter) in the non-HT group (11/38, 28.9%) and none in the HT group (28.9% vs. 0.0%, p = 0.023). Compared with the non-HT group, the HT group was significantly associated with higher incidences of extrathyroidal extension (47.4% vs. 85.7%, p = 0.013) **Figures 1A–D**) and capsular invasion (71.1% vs. 100.0%, p = 0.023). On the other hand, there were no significant differences in age, sex, multifocal, TNM staging, T staging, N staging, and BRAF^V600E^ mutation status (all p < 0.05).

### 3.2 Ultrasonographic features

**Figure 2:**
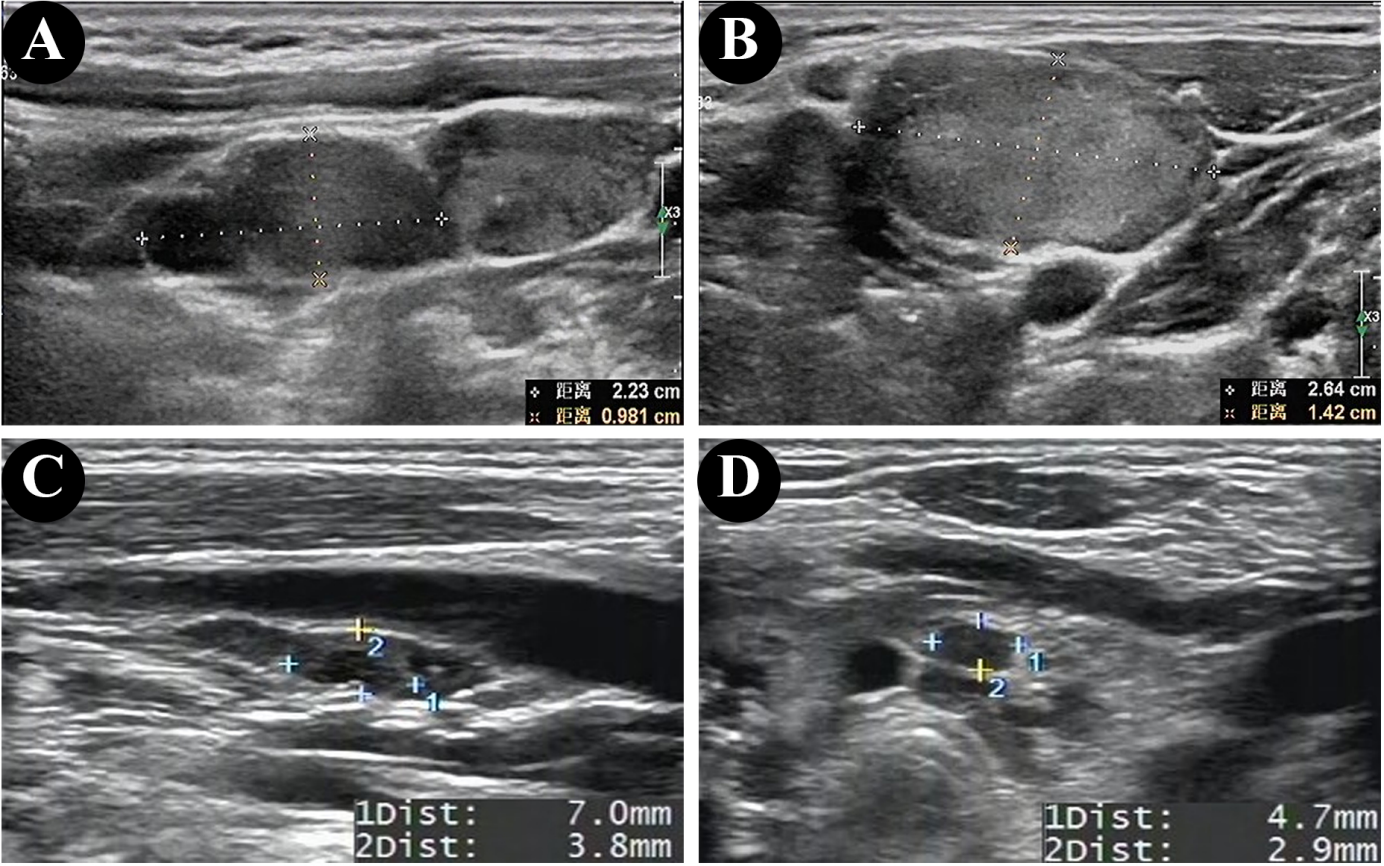
Ultrasonographic findings of lateral lymph node metastasis in patients without HT **(A, B)** and central lymph node metastasis in patients with HT **(C, D)**.

**Figure 3:**
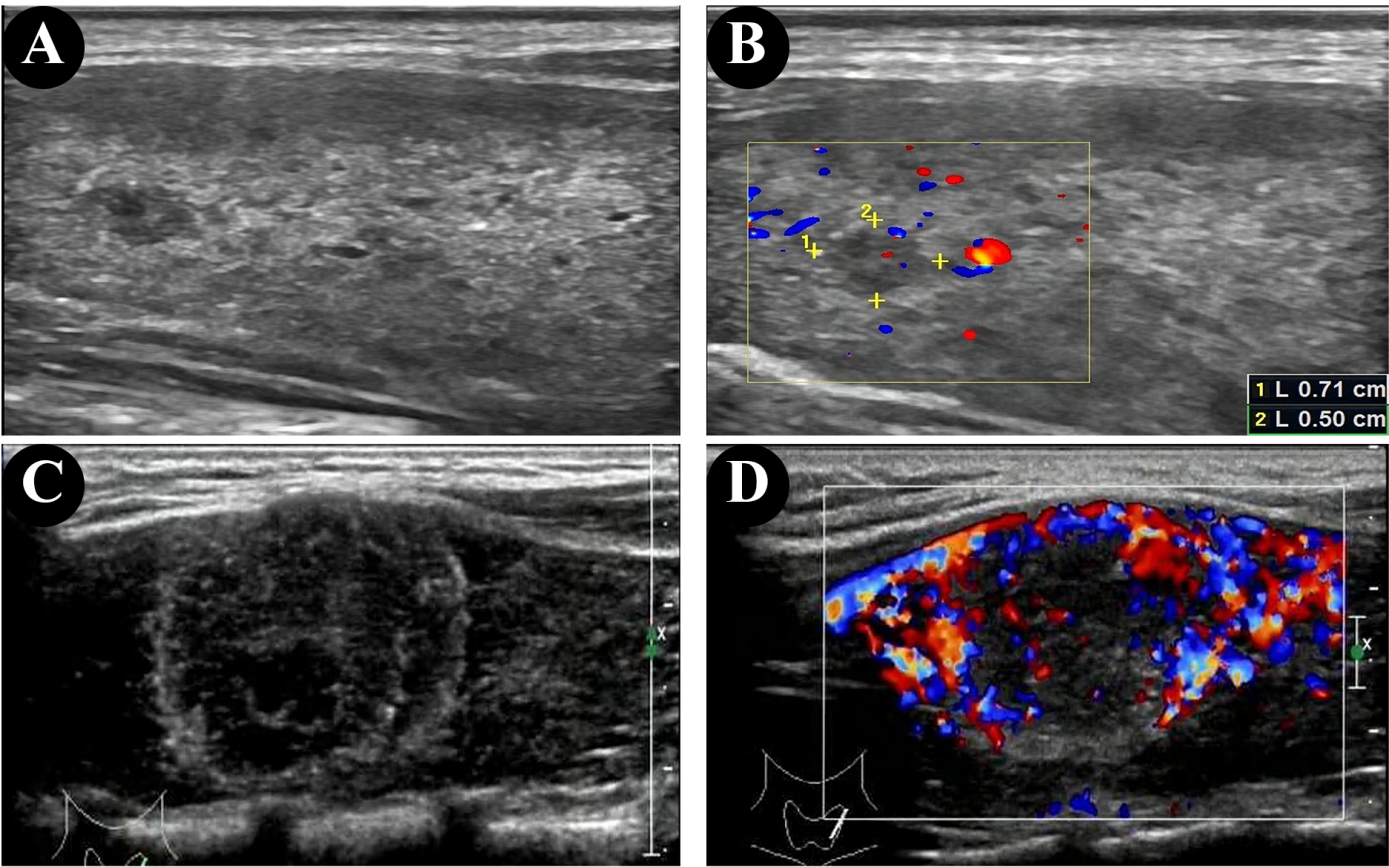
Ultrasonographic findings of papillary thyroid microcarcinomas (PTMC) in the patient without HT **(A, B)** and punctate echogenic foci in the patient with HT **(C, D)**.

The ultrasonographic features of patients with and without HT are shown in **Table 2**. In both groups, most of the patients presented with typical PTC characteristics such as solid or almost completely solid nodules, marked hypo-echogenicity, irregular shape, irregular margins, PEF, and lateral neck lymph node metastasis (LLNM). Among patients with PTC and HT, 92.9% (13/14) had LNM (**Figures 2C, D**), 50.0% (7/14) had central lymph nodes metastasis (CLNM) only, and 42.9% (6/14) had both CLNM and LLNM; among patients without HT, 63.2% (24/38) had LNM (**Figures 2A, B**), 26.3% (10/38) had CLNM only, and 36.8% (14/38) had both CLNM and LLNM, with a statistically significant difference (p = 0.036). There were no differences between the groups regarding composition, echogenicity, shape, margin, TI-RADS categories, and total points. The frequency of PEF in patients with PTC and HT (**Figures 3C, D**) was significantly higher than that in patients with PTC and HT alone (p = 0.016) (**Figures 3A, B**).

**Table 2:** Ultrasonographic features between children and adolescent patients with papillary thyroid carcinoma with or without Hashimoto’s thyroiditis (HT).

Ultrasonographic	PTC with HT	PTC without TH	p
Number of patients	14	38	
Composition			
Cystic and mixed	0	1	0.534
Solid or almost completely solid	14	37	
Echogenicity			
Hyperechoic or isoechoic	0	7	0.843
Hypoechoic	14	31	
Shape			
Wider-than-tall	14	37	0.534
Taller-than-wide	0	1	
Margin			
Smooth	0	2	0.167
Ill-defined	1	11	
Lobulated or irregular	9	13	
Extra-thyroidal extension	4	12	
Echogenic foci			
None or large comet-tail artifacts	0	11	0.016
Macrocalcifications	0	1	
Punctate echogenic foci	14	26	
Lymph node metastasis			
No LN metastasis	1	14	0.036
LN metastasis (central/lateral)	13(7/6)	24(10/14)	
TI-RADS categories			
TR 3	0	5	0.219
TR 4	1	6	
TR 5	13	27	
Total points			
Mean ± SD	9.21 ± 1.63	7.79 ± 2.65	0.066
Range	4-11	3-12	

## 4 Discussion

Lindsay et al. (17) first reported the association between HT and PTC in 1952; however, to date, the relationship between these two disorders remains complex and not completely understood (18, 19). Patients with HT are at an increased risk of PTC compared with the general population (20), some studies showed HT increased the risk of PTC (21, 22). There are few reports on the ultrasonographic features of PTC in children and adolescent patients with HT; moreover, the association between PTC and HT has been poorly defined in China. Therefore, we herein retrospectively analyzed the ultrasonographic, clinical, and histopathologic features of children and adolescent patients with PTC with and without HT.

A total of 52 children and adolescent patients with PTC who underwent thyroidectomy were included; 14 patients were diagnosed with HT confirmed by pathology. The prevalence rate of PTC in children and adolescent patients with HT was 27% in the thyroidectomy specimens obtained from our patients, which was similar to the findings from a previous systematic meta-analysis that reported a PTC prevalence rate of 33% among patients with HT (20). These incidences are comparable to the worldwide prevalence of PTC of approximately 23% (10%–58%) in adult patients with HT (23).

The relationship between HT and thyroid cancer may be related to chronic inflammation and autoimmune dysfunction. Chronic inflammation will lead to the release of reactive oxygen species and inflammatory cytokines, inducing cell proliferation, cell repair, and the formation of a chronic inflammatory environment (24). Reactive oxygen species produced in an inflammatory environment can cause DNA damage in organs, which is a common mechanism of cancer development (25). Autoimmune dysfunction may lead to the immune escape of tumor cells, making it further difficult for the tumor cells to be detected and cleared by the immune system, thus promoting the development of tumor (26). However, Radetti et al. (27)concluded that HT may impact the development of thyroid nodules but not cancer in pediatric patients. This is different from our study’s conclusion. The probable reason is that Radetti et al. studied the development of HT patients who had no nodules from the beginning and we studied the cancer biology behavior of diagnosed PTC. Up to now, whether pre-existing HT should be considered a high-risk factor for PTC development in pediatric patients remains unclear. More studies on the association between HT and the risk of PTC are needed (28).

Most of the studies on the potential association between PTC and HT development have been conducted in adults; reports on this association in children and adolescents are rare. Therefore, we compared the ultrasonographic, clinical, and pathological features of children and adolescents with PTC with and without HT.

The results of the present study showed that the percentage of papillary thyroid microcarcinomas was higher in the non-HT group than in the HT group and that the rate of extrathyroidal extension and capsular invasion was higher in patients with HT than in those with PTC alone, which were consistent with the findings of our previous study (12, 29). Patients with thyroid cancer who have an extrathyroidal extension and capsular invasion are considered to have more advanced tumors. Our present results showed that the presence of HT is associated with a bigger tumor size and stronger invasion ability and that the rate of LNM was more frequent in patients with HT and PTC than in patients with PTC alone, which was similar to our previous findings that extrathyroidal extension is strongly associated with LNM (30).

HT is the most common form of thyroiditis, a condition characterized by diffuse lymphocytic infiltration, gradual destruction of the gland, and fibrosis (31). Histologically confirmed HT is considered an independent risk factor and presents a higher incidence of PTC in children and adolescents (32, 33). The results of the present study showed that the positive rate of LNM is significantly higher in patients with PTC and HT than in patients with PTC alone, consistent with the findings of a previous study (29). However, there is disagreement with the point of view that HT in children and adolescents with PTC does not affect LNM (34). For instance, a cross-sectional study evaluated the differences between 106 children and adolescents with PTC and 23 patients with PTC and HT and reported that 16 (69.6%) and 10 (43.5%) patients were separately positive for CLNM and LLNM with PTC and HT compared with 67 (63.2%) and 50 (47.2%) patients with PTC alone. There were no differences between the two groups in terms of LNM (32). In the present study, among patients with PTC and HT, 92.9% (13/14) had LNM, 50.0% (7/14) had central lymph nodes metastasis (CLNM) only, and 42.9% (6/14) had both CLNM and LLNM; among patients without HT, 63.2% (24/38) had LNM, 26.3% (10/38) had CLNM only, and 36.8% (14/38) had both CLNM and LLNM, with a statistically significant difference (p = 0.036). This finding suggests that the coexistence of HT and PTC should be considered a higher risk for LNM in children and adolescents.

PEF are useful in diagnosing PTC and predicting the aggressiveness of PTC (35); our study showed a higher detection rate (100%) of PEF in the HT group than in the non-HT group (68.4%). In the 2017 ACR TI-RADS, PEF is assigned 3 points, associated with a high suspicion of malignancy (15). Middleton et al. (36) reported that the risk of malignancy was 29.8%–40.5% in solid nodules and 5.9%–15.1% in nodules mixed with PEF. PEF in thyroid nodules is considered as predictor of malignant thyroid cancer in children and adolescents (35, 37, 38). The cause of PEF is related to the rapid growth of cancer cells and insufficient blood supply to tissues, followed by degeneration, necrosis, and calcium deposition (39–41). It is commonly held that small echogenic foci without sound shadow on ultrasonographic features, often termed microcalcifications or psammoma bodies in thyroid tumors (38). However, in a few cases, the rear sound shadow can be generated due to the aggregation of multiple PEF (42).

Due to the high invasiveness of HT and PTC, surgical techniques, including lobectomy, total or near-total thyroidectomy, whether they have peripheral lymph node dissection, need to be comprehensively evaluated. The collaborative diagnosis and treatment of multidisciplinary teams are crucial. It could guarantee the precise treatment of thyroid cancer, thus improving the prognosis.

Nevertheless, there are some potential limitations to our study, including the retrospective study design and the potential for bias in diagnostic coding. Moreover, the number of cases encountered in a single institution was very small; therefore, these factors must be carefully considered when interpreting the findings of our study. Moreover, the lateral neck region was selectively dissected; therefore, a few LNMs might have been missed *via* LN staining. However, the BRAF^V600E^ mutation status was detected in 42 out of 52 PTC cases, and not all patients, further increasing the bias of the associations of HT with the gene of BRAF^V600E^ in this study.

The serum thyroid antibodies of all patients in the HT group were listed in **Supplementary Table 1**. The serum antibodies result of patient 3 and patient 4 do not increase. Radetti et al. reported that 10–15% of patients with HT will be negative for thyroid antibodies, normal serum thyroid level does not exclude the diagnosis as in many cases of thyroiditis the function may be perfectly normal (43). And some studies implied that the presence of lymphocytes in contact with thyroid cells is considered the most important element to make a differential diagnosis between HT and thyroid tumors (6, 43). T lymphocytes are the major subgroup of tumor-infiltrating immune cells, among which CD8+ T cells and CD4+ T cells comprise the primary immune cells responsible for anti-tumor immunity. Wang et al. showed that CD4+ and CD8+ TILs infiltration in PTC with HT tissues were significantly higher than in PTC tissues (44). Pan et al. showed that the immune cells in tumors exhibited distinct transcriptional states, and the presence of tumor-infiltrating B lymphocytes was predominantly linked to concurrent HT origin. Trajectory analysis of B cells and plasma cells suggested their migration potential from HT adjacent tissues to tumor tissues (45). And HT presents diffuse lymphocytic infiltration while tumor presents partly lymphocytic infiltration. The pathology results showed that these 14 patients had diffuse lymphocytic infiltration. We have insulted pathologists in our institute and made an agreement that HT can be diagnosed by pathology. Here in our study, post-surgery pathology has proved whether patient suffered HT.

In addition, a control group such as patients with benign disease with and or Hashimoto’s thyroiditis is needed in the prospective study of the association of PTC and HT, which can decrease the bias and collect enough data to analyze, which makes the results more persuasive.

Finally, we just compared the ultrasonographic, clinical, and histopathological features of children and adolescent patients with PTC and HT with those of patients without HT. Future studies might also need to include other aspects, such as thyroid autoimmune antibodies (TGAb and TPOAb), PTC types, and the genes related to thyroid carcinoma, control group to construct large sample research.

In conclusion, our results suggest that HT may be an independent risk factor in children and adolescents with PTC, which showed more aggressive features, including larger tumor size, higher rate of extrathyroidal extensions, capsular invasion, PEF, and LNM compared with PTCs in patients without HT.

## Data availability statement

The original contributions presented in the study are included in the article/**Supplementary Material**. Further inquiries can be directed to the corresponding author.

## Ethics statement

Approval for this retrospective study was obtained from the Institutional Research Ethics Board of Sun Yat-sen Memorial Hospital, Sun Yat-sen University, Guangzhou, China; the requirement for informed patient consent was waived.

## Author contributions

RL and JR designed the study. YJ and ML reviewed the literature, analyzed data, and drafted the manuscript. All authors read and approved the final manuscript.
