# Supplementary material for: Ultrasonographic, clinical, and pathological features of papillary thyroid carcinoma in children and adolescents with or without Hashimoto’s thyroiditis
Source: Front Oncol. 2023 Aug 1;13:1198468. doi: 10.3389/fonc.2023.1198468 (PMC10428624; doi:10.3389/fonc.2023.1198468)
Supplement: Supplementary file 1 [file Table_1.doc]

| Patient | Sex | Age | T3  (nmol/L) | T4  (nmol/L) | TSH  (mU/L) | Anti-thyroid peroxidase antibody  (U/mL) | Anti-thyroglobulin antibody  (IU/mL) |
| --- | --- | --- | --- | --- | --- | --- | --- |
| 1 | F | 16 | 5.63 | 16.38 | 3.994 | 1867 | 315 |
| 2 | F | 15 | 5.4 | 18.24 | 1.65 | ＞1300 | ＜15.0 |
| 3 | F | 8 | 5.9 | 20.06 | 1.53 | ＞1300 | 23 |
| 4 | F | 17 | 4.4 | 17.54 | 9.96 | ＞1300 | 24 |
| 5 | F | 12 | 6.4 | 17.28 | 0.03 | ＜28.0 | 17 |
| 6 | F | 17 | 4.21 | 17.54 | 0.454 | ＞1300 | 26 |
| 7 | M | 15 | 5.8 | 10.3 | 0.01 | ＞1300 | ＜15.0 |
| 8 | F | 17 | 4.9 | 15.63 | 1.76 | ＜28.0 | 268 |
| 9 | F | 13 | 6.41 | 19.7 | 0.954 | 6409 | 32 |
| 10 | F | 17 | 4.6 | 13.4 | 0.85 | ＞1300 | ＜15.0 |
| 11 | F | 16 | 5.7 | 19.09 | 1.51 | 99 | 163 |
| 12 | F | 15 | 5.99 | 18.12 | 1.612 | 58 | 395 |
| 13 | F | 15 | 5.7 | 16.96 | 0.48 | 887 | ＜15.0 |
| 14 | F | 18 | 2.9 | 2.9 | ＞150 | ＞1300 | ＜15.0 |

**Supplementary Table 1. Serum Thyroid Antibodies level of the HT group**
