# Supplementary material for: Ultrasonographic, clinical, and pathological features of papillary thyroid carcinoma in children and adolescents with or without Hashimoto’s thyroiditis
Source: Front Oncol. 2023 Aug 1;13:1198468. doi: 10.3389/fonc.2023.1198468 (PMC10428624; doi:10.3389/fonc.2023.1198468)
Supplement: Supplementary file 2 [file Table_2.docx]

**Supplementary Table 2. Serum Thyroid Antibodies level of the non-HT group**

| Patient | Sex | Age | T3  (nmol/L) | T4  (nmol/L) | TSH（mU/L） | Anti-thyroid peroxidase antibody （U/mL） | Anti-thyroglobulin antibody（IU/mL） |
| --- | --- | --- | --- | --- | --- | --- | --- |
| 1 | F | 13 | 5.8 | 14.96 | 0.87 | ＜28.0 | 22 |
| 2 | F | 14 | 5 | 18.86 | 0.59 | ＜28.0 | ＜15.0 |
| 3 | F | 12 | 6.3 | 14.26 | 1.88 | / | 125 |
| 4 | F | 14 | 5.09 | 16.84 | 3.454 | 4004 | ＜15.0 |
| 5 | F | 9 | 6 | 18.61 | 2.58 | 30 | ＞500 |
| 6 | F | 12 | 4.3 | 14.72 | 0.25 | ＜28.0 | 32 |
| 7 | F | 14 | 5.2 | 23.33 | 4.64 | ＜28.0 | ＞2500 |
| 8 | F | 18 | 3.5 | 7.38 | 4.31 | ＞1300 | 496 |
| 9 | M | 15 | 5 | 16.04 | 1.02 | ＜28.0 | 125 |
| 10 | M | 14 | 6.8 | 17.04 | 1.15 | 33 | 36 |
| 11 | F | 17 | 3.1 | 3.57 | 0.52 | ＞1300 | ＞500 |
| 12 | F | 16 | 5.7 | 18.9 | 0.43 | ＜28.0 | 24 |
| 13 | F | 17 | 1.4 | 93.1 | 2.91 | 73 | ＜15.0 |
| 14 | F | 10 | 6 | 14.62 | 1.95 | 223 | ＜15.0 |
| 15 | F | 15 | 4.8 | 16.3 | 1.9 | 29 | **> 500.0** |
| 16 | M | 16 | 6.6 | 16.87 | 1.3 | ＜28.0 | ＜15.0 |
| 17 | M | 17 | 11.2 | 41.76 | 0.02 | ＞1300 | ＞500 |
| 18 | F | 13 | 6.9 | 16.29 | 0.6 | ＜28.0 | / |
| 19 | F | 18 | 4.6 | 18.02 | 0.05 | 44 | 157 |
| 20 | F | 18 | 4.4 | 12.98 | 3.26 | ＜28.0 | 126 |
| 21 | F | 16 | 4.7 | 18.57 | 0.82 | 38 | 17 |
| 22 | F | 17 | 9.9 | 15.81 | 0.01 | ＞1300 | ＜15.0 |
| 23 | F | 11 | 5.9 | 21.11 | 4.96 | ＞1300 | ＜15.0 |
| 24 | F | 17 | 4.7 | 15.48 | 2.92 | ＜28.0 | 29 |
| 25 | F | 17 | 4.9 | 17.36 | 1.19 | ＜28.0 | ＞500 |
| 26 | F | 18 | 5.69 | 27.77 | 0.078 | ＜28.0 | ＜15.0 |
| 27 | F | 18 | 1.71 | 89.3 | 1.301 | 33 | ＞2500 |
| 28 | M | 11 | 6.8 | 17.8 | 2.34 | ＜28.0 | 53 |
| 29 | M | 14 | 5.6 | 13.43 | 2.09 | 36 | ＜15.0 |
| 30 | M | 9 | 6.2 | 18.57 | 2.42 | 32 | 55 |
| 31 | F | 17 | 3.4 | 9.89 | 0.02 | ＜28.0 | 64 |
| 32 | F | 12 | 5.6 | 15.93 | 2.08 | 44 | ＜15.0 |
| 33 | M | 17 | 5.3 | 17.1 | 1.27 | ＜28.0 | 38 |
| 34 | F | 17 | 9.5 | 30.03 | 0.01 | ＜28.0 | ＜15.0 |
| 35 | M | 4 | 6.5 | 21.17 | 4.26 | ＜28.0 | 42 |
| 36 | F | 14 | 5.1 | 14.9 | 2.39 | 36 | 39 |
| 37 | F | 16 | 4.7 | 13.15 | 1.16 | ＜28.0 | 249 |
| 38 | F | 8 | 6 | 16.37 | 2.79 | 29 | ＞500 |
